# Hyaluronidase expression within tumors increases virotherapy efficacy and T cell accumulation

**DOI:** 10.1016/j.omto.2021.05.009

**Published:** 2021-05-29

**Authors:** Martí Farrera-Sal, Rafael Moreno, Ana Mato-Berciano, María Victoria Maliandi, Miriam Bazan-Peregrino, Ramon Alemany

**Affiliations:** 1 ProCure Program, IDIBELL-Institut Català d’Oncologia, l’Hospitalet de Llobregat, Barcelona 08908, Spain; 2 VCN Biosciences S.L., Sant Cugat del Vallès, Barcelona 08174, Spain

**Keywords:** oncolytic, adenovirus, virotherapy, cancer, immunotherapy, hyaluronidase, hyaluronan, T-cell infiltration

## Abstract

Oncolytic viruses (OVs) preferentially infect and selectively replicate in cancer cells. OVs have been tested in clinical trials as monotherapy or in combination with chemotherapy, radiotherapy, and immunotherapy. However, the dense extracellular matrix hampers the intratumoral spreading and efficacy of OVs. Previously we described VCN-01, an oncolytic adenovirus expressing a soluble version of human sperm hyaluronidase (hyal) PH20, which exhibited enhanced intratumoral distribution and antitumor activity in different models. Here, we present two oncolytic adenoviruses designed to increase the secretion of PH20 compared to VCN-01. ICO15K-40SAPH20, encoding PH20 under an Ad40 splice acceptor, and ICO15K-E1aPH20 expressing PH20 fused to the *E1A* gene by P2A peptide. We demonstrate that increased hyal activity improves antitumor efficacy in both a sensitive immunodeficient model and an immunocompetent model. Moreover, we show that hyal activity impacts T cell accumulation in tumors, highlighting the value of a hyaluronidase-expressing virus for combinations with other immunotherapies in cancers involving dense stroma.

## Graphical abstract

**Figure:**
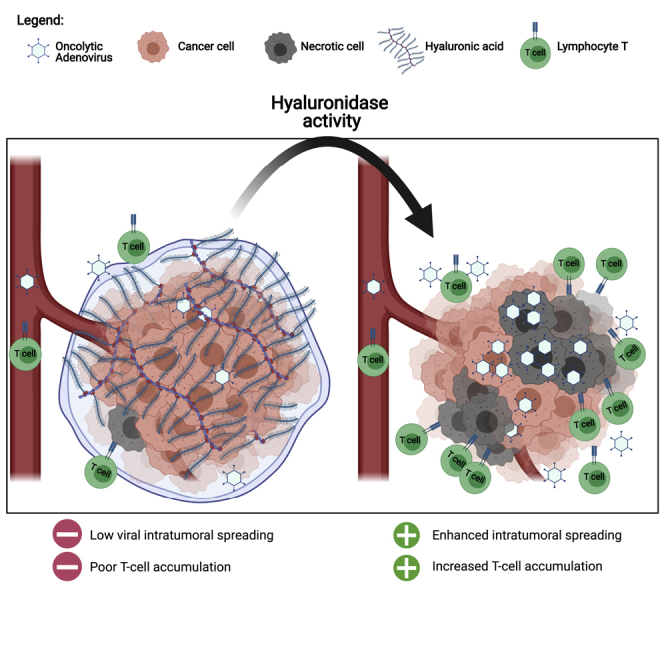

## Teaser

Oncolytic viruses expressing hyaluronidase have improved intratumoral spreading and efficacy. Here, Farrera-Sal et al. demonstrated that an increase of the hyaluronidase activity enhances the antitumor effect and could be potentially combined with other immunotherapies increasing the T cell accumulation into treated tumors.

## Introduction

Solid tumors are complex organ-like structures consisting of cancer cells, vasculature, extracellular matrix (ECM), stromal, and immune cells. One of the main ECM components is hyaluronic acid (HA), which accumulates in many solid tumors, including pancreatic ductal adenocarcinomas,^1^ breast, colon, and prostate cancer,^2^ among others. HA is associated with immunosuppression, metastatic potential, and poor prognosis.^3^, ^4^, ^5^, ^6^ Moreover, HA retains water molecules thereby increasing tumor interstitial pressure, which plays an important role in resistance to drugs’ extravasation.^7^^,^^8^

Oncolytic viruses (OVs) have the ability to selectively replicate in cancer cells without harming normal tissues.^9^^,^^10^ OVs lyse tumor cells and trigger a pro-inflammatory response that may induce antitumor immunity, making them attractive for immunotherapy combinations.^11^ However, their intratumoral spread is hampered by the ECM, which acts as a physical barrier for viral distribution.^12^ ECM-degrading enzymes are commonly exploited to enhance viral penetration in solid tumors.^13^ Our group generated a hyaluronidase-expressing oncolytic adenovirus (OAd), called VCN-01.^14^ VCN-01 has exhibited a favorable toxicity profile and potent antitumor efficacy in different models of cancer.^14^, ^15^, ^16^, ^17^ VCN-01 is currently under clinical trial investigation to treat advanced pancreatic cancer (NCT02045602, NCT02045589), retinoblastoma (NCT03284268),^16^ and head and neck cancers (NCT03799744). While the results of VCN-01 are promising, in this work we hypothesize that increased levels of hyaluronidase (hyal) expression may improve oncolytic activity. Accordingly, we generated OAds with higher hyal activity to assess the impact of HA degradation on antitumor efficacy and immune response.

## Results

### Generation of OAds with enhanced hyal activity compared to VCN-01 and preserved oncolytic properties

**Figure 1:**
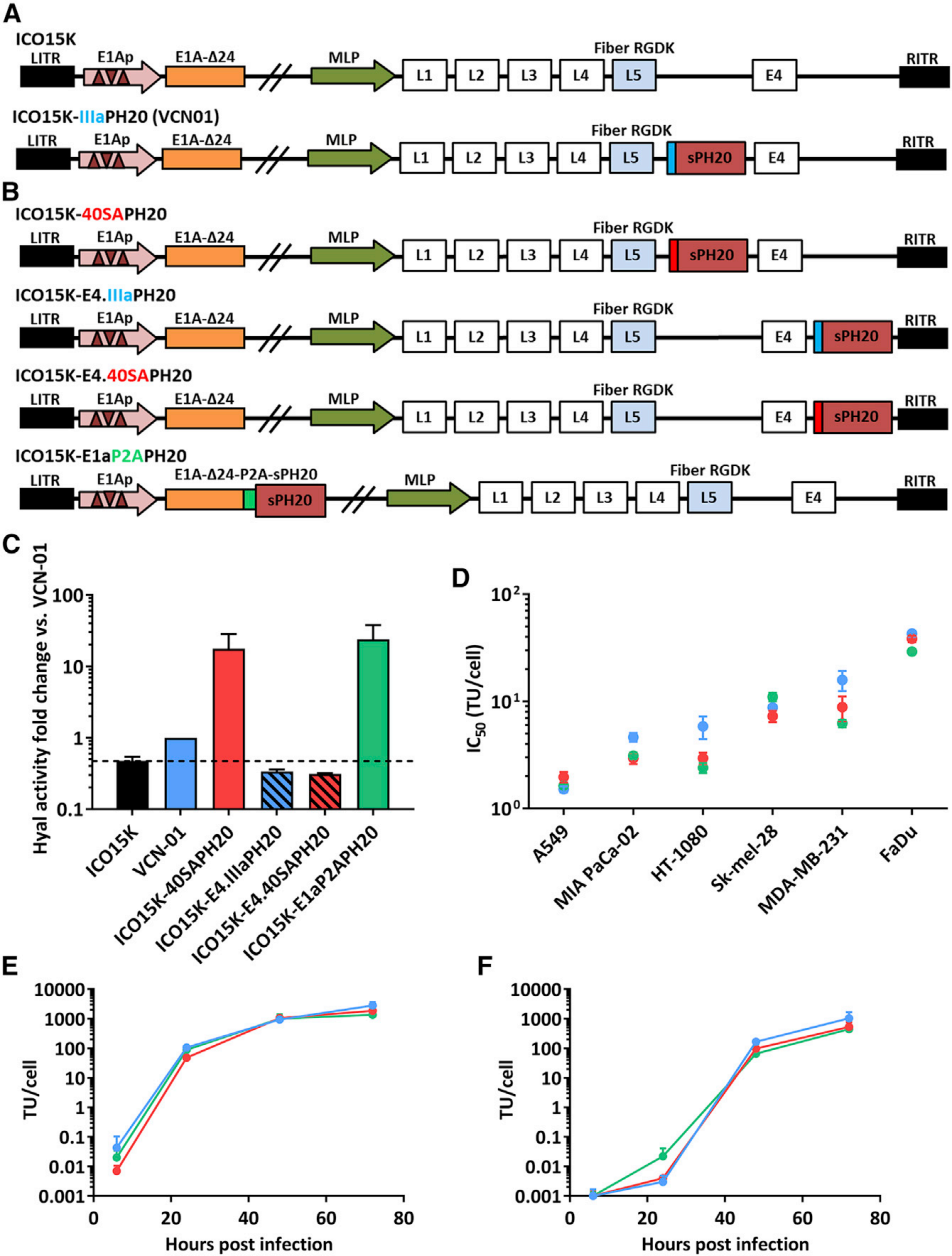
Selection of Hyal-OAds (A) ICO15K carries the *E1A*Δ24 mutation and E2F binding sites in the promoter of the gene (represented as brown triangles), and the RGDK motif in the fiber shaft also known as a RGDK fiber. ICO15K was used to introduce a soluble version of human PH20 (sPH20) after fiber (Late 5 gene, L5) under IIIa splice acceptor (represented in blue) restricted to major late promoter (MLP) generating ICO17K or VCN-01. (B) ICO15K was engineered to generate all hyaluronidase (hyal)-expressing viruses. sPH20 was inserted after fiber under Ad40 long fiber SA (40SA, represented in red), or downstream E4 gene with IIIa or 40SA splice acceptors and fused with P2A sequence (represented in green) to the *E1A* gene. (C) A549 cells were infected at MOI 20 for 72 h and SNs were harvested and concentrated 30-fold to assess the hyal activity by a turbidimetric assay. The fold change of hyal-activity versus VCN-01 (positive control) is represented. Mean of triplicates ± SEM is plotted; the dotted line indicates the hyal activity threshold set by ICO15K (negative control). (D) A dose-response cytotoxic assay was performed *in vitro* in a panel of human cancer cells 96 h after infection: lung adenocarcinoma (A549), pancreas carcinoma (MIA Paca-02), connective tissue fibrosarcoma (HT-1080), melanoma (Sk-mel-28), breast adenocarcinoma (MDA-MB-231), and pharynx squamous cell carcinoma (FaDu). The mean of IC_50_ triplicates ± SD is represented and assessed by Kruskal-Wallis with Dunn’s post hoc test. (E and F) Total virus production in (E) culture cell extracts (CEs) or in (F) SNs. The A549 cell line was infected at MOI 20 for 4 h. Then, virus excess was washed and cells were incubated for 24, 48, or 72 h. CEs and SNs were collected, and virus production was determined using anti-hexon staining. Results are expressed as transducing units (TUs) produced per cell. Mean ± SD of triplicates is shown.

We have previously reported the generation of OAd ICOVIR15K^18^ (abbreviated here as ICO15K) and VCN-01 (also known as ICOVIR17K or ICO17K) expressing a soluble version of the human PH20 hyal under IIIa splice acceptor^19^ (Figure 1A). To obtain viruses with higher hyal activity, we designed and generated four new hyal-expressing OAds (hyal-OAds; Figure 1B). First, the transgene splice acceptor IIIa was replaced with a previously reported strong splice acceptor from the long fiber gene of Adenovirus 40, known as 40SA splicing acceptor,^20^ generating ICO15K-40SAPH20. Then, to test different genomic locations, the PH20 was introduced downstream of the *E4* unit with the two splice acceptors, obtaining ICO15K-E4.IIIaPH20 and ICO15K-E4.40SAPH20. In another strategy, hyal was expressed as an early gene fused to *E1A* by means of the self-cleavable peptide P2A^20^ to generate ICO15K-E1aPH20.

The hyal activity of the new hyal-OAds was assessed in A549 cells as a reference cell line for adenovirus infection after 72 h. The supernatants (SNs) were harvested and concentrated to perform a HA degradation assay (turbidimetric assay). The viruses with hyal downstream of E4 did not show any transgene activity. ICO15K-E1aPH20 and ICO15K-40SAPH20 showed higher hyal activity than VCN-01 (Figure 1C). In line with these results, ICO15K-40SAPH20 and ICO15K-E1aPH20 were selected for further development, having obtained comparable titers to VCN-01 (Table S1).

To focus on the role of hyal expression, viruses with cytotoxic properties similar to VCN-01 were needed. We addressed the oncolytic potential of ICO15K-40SAPH20 and ICO15K-E1aPH20 compared to VCN-01 by employing dose-response cytotoxic assays in a panel of cancer cell lines. Cytotoxicity was first evaluated in A549, yielding comparable oncolytic potency (IC_50_ values) among the selected viruses. Then, six cancer cell lines were tested without significant differences in IC_50_ between ICO15K-40SAPH20, ICO15K-E1aPH20, and VCN-01 (Figure 1D). Of note, the IC_50_ values observed in the Sk-mel-28 model are particularly similar.

As another parameter of virus fitness, we assessed the virus production kinetics of the new viruses in A549 by measuring the infective viral yields (transfecting units, TU) released to SNs and inside the cell (also called cell extract, CE). No significant differences in total virus production were found among the different viruses compared to VCN-01 (Figures 1E and 1F). Though not statistically significant, it is worth mentioning that ICO15K-E1aPH20 produced the lowest amount of TU per cell (Figure S1A).

### Effect of enhanced hyal activity expressed by oncolytic adenoviruses in tumors

**Figure 2:**
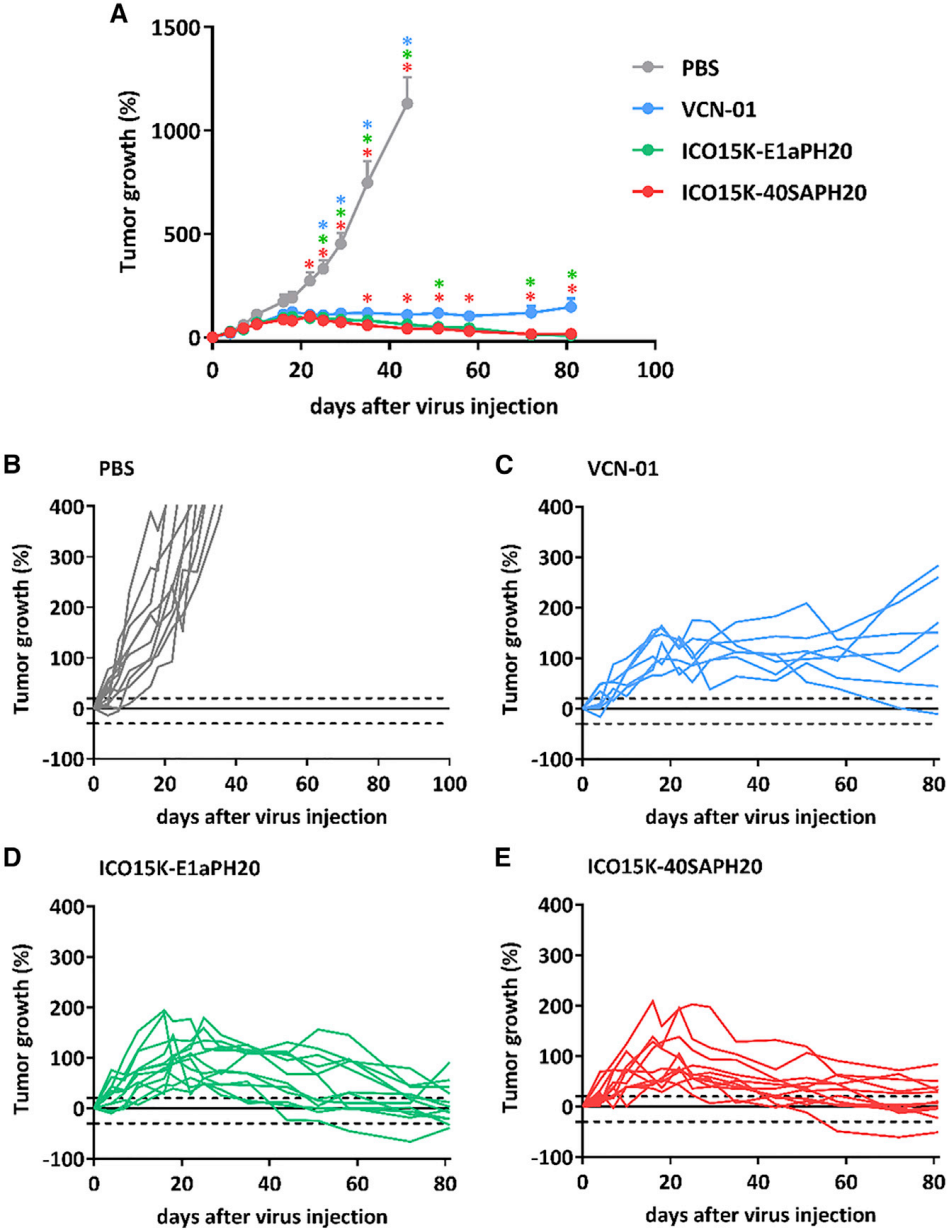
Antitumor efficacy of Hyal-OAds in Sk-mel-28 *in vivo* (A) Sk-mel-28 tumors implanted in NSG mice were treated (2e10 vp/animal intravenously) at a mean volume of 180 mm^3^ and monitored for 81 days. Mean tumor growth is represented + SEM. ∗p < 0.05 significant versus corresponding group (color) by two-way ANOVA and Tukey post hoc test. (B–E) Detailed tumor growth of treated tumors from the same experiment. Dotted lines on y axis at 20% and −30% indicate the criteria for clinical status. Progressive disease ≥ 20%, stable disease < 20% to > −30%, partial response ≤ −30%, complete response = −100%.

The Sk-mel-28 model was selected to test the efficacy of the hyal-expressing viruses, as we previously reported that these tumors are rich in HA content.^21^ Moreover, we confirmed the hyal activity in this model in a pilot *in vivo*. The lack of HA staining near replication sites (E1A protein staining) in Sk-mel-28 treated tumors confirmed the PH20 activity of VCN-01 and ICO15K-40SAPH20 (Figure S2). To assess efficacy, we subcutaneously implanted NSG mice with Sk-mel-28 tumors and treated them intravenously with 2 × 10^10^ vp per animal. After 25 days of treatment, all viruses presented significant antitumor activity compared to PBS (Figure 2A). After day 35, increased tumor growth control of ICO15K-40SAPH20 and ICO15K-E1aPH20 compared to VCN-01 was observed. ICO15K-40SAPH20 and ICO15K-E1aPH20-treated groups regressed almost to initial tumor volumes (tumor growth 15% and 8% at the endpoint, respectively), while the VCN-01 group maintained a slow but sustained tumor growth (146%; Figures 2C–2E). At the endpoint, macroscopic observations indicated that some tumors were mainly necrotic and fibrotic areas, whereas some tumors presented viable growing tumor nodules. VCN-01-treated tumors had more viable nodules (Figure S3A) than other treated groups. These macroscopic observations were confirmed with hematoxylin and eosin staining (H&E) to identify viable zones (blue staining of the nucleus) and non-cellular zones (red staining, Figures S3B and S3C). The new hyal-OAds showed approximately 7% tumor viability at the endpoint compared to 17% of VCN-01 (Figure S3D). The results also indicate a greater antitumor efficacy than estimated by tumor volume.

Hyal-OAds had significant long-term control of xenograft models in immunodeficient mice. With the aim of assessing the relevance of hyal expression for the immune response against the tumor, we used an immunocompetent mouse model. However, human adenoviruses do not replicate in most murine cell lines;^22^ they poorly translate late virus genes and transgenes in late transcription units.^23^ Taking this into account, we evaluated virus production in the semi-permissive mouse cell line CMT64.6, generated by our group.^24^ Virus production was around 4 TU per infected cell for all viruses at 72 h post-infection (Figures S4A and S4B). Considering that the IC_50_ values in this model ranged from 75 TU/cell to 49 TU/cell after 4 days of infection, progression of the cytopathic effect was clearly limited (Figure S4C). Consistent with the restricted expression of late viral genes, hyal activity was detected only in ICO15K-E1aPH20 supernatants, as PH20 expression in this virus was linked to the early gene *E1A* (Figure S4D). Thus, we considered VCN-01 as a minimal hyal-expressing virus and ICO15K-E1aPH20 as a high hyal-expressing virus in this model.

**Figure 3:**
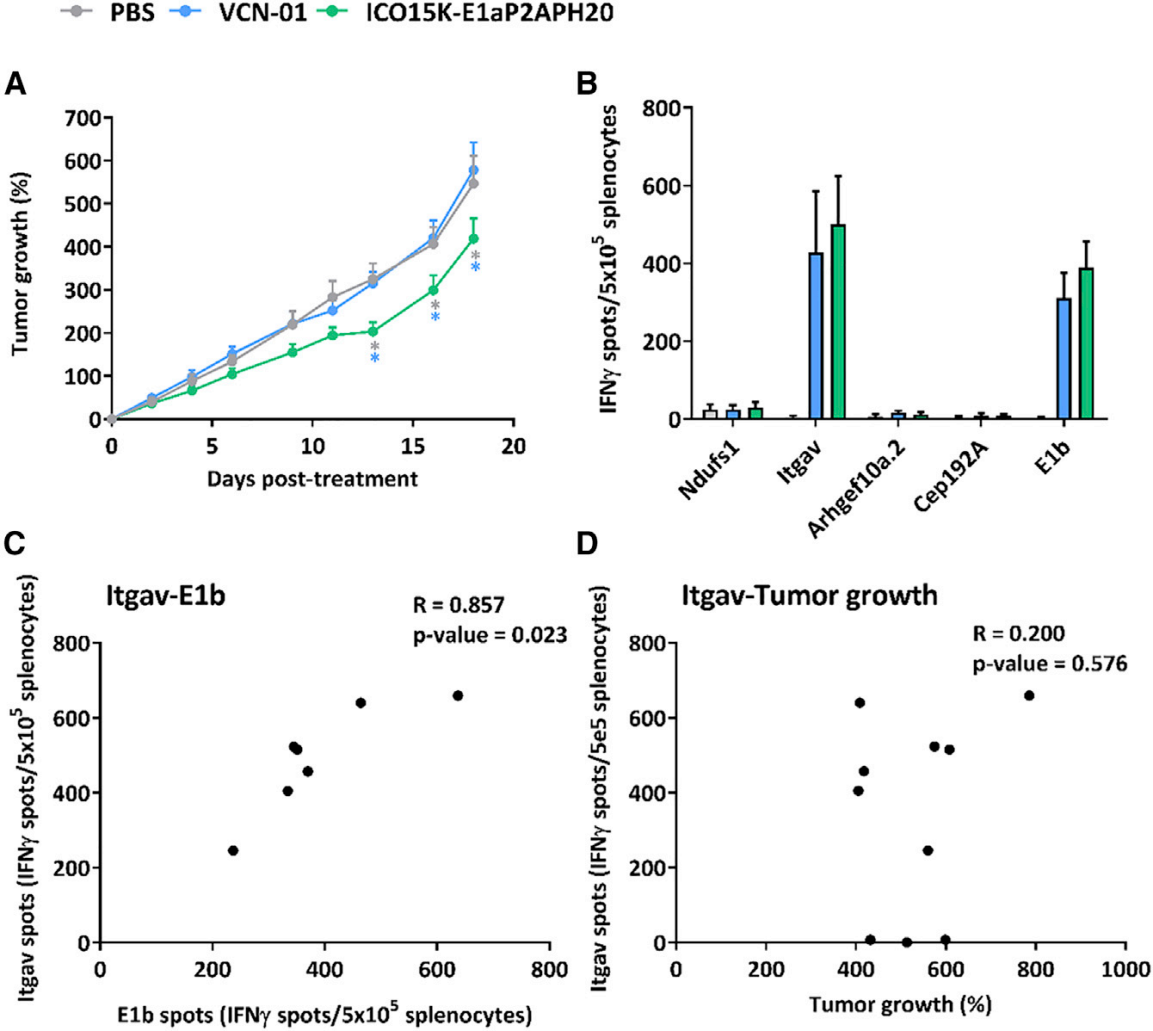
Hyal expression is crucial for *in vivo* efficacy against CMT64.6 tumors (A) Tumor growth of CMT64.6 tumors was monitored every 3–4 days until the end of the study (each group had n ≥ 10 tumors). The mean + SEM is plotted. ∗p < 0.05 versus other groups (colors) by two-way ANOVA and Tukey’s post hoc test. (B) Spleens were collected (n ≥ 3/group) to perform an ELISpot against 4 CMT64.6 neoepitopes and the E1b viral epitope. Mean + SD of IFN-γ spots with background subtraction is plotted. (C) Spearman correlation between Itgav spots and E1b spots in VCN-01 and ICO15K-E1aPH20 groups together (n = 7). (D) There was no correlation between Itgav spots and the % of tumor growth at the endpoint (n = 10). The mean of duplicate with background value subtracted is shown for each animal in each correlation.

We tested the efficacy of VCN-01 and ICO15K-E1aPH20, treating C57BL/6 mice bearing CMT64.6 tumors. Animals were injected with 1 × 10^9^ TUs intratumorally to maximize virus presence in tumors. ICO15K-E1aPH20 presented a statistically delayed tumor growth after day 13, compared to PBS and VCN-01 (Figure 3A). At the end of the experiment, spleens were harvested, and an enzyme-linked immunosorbent assay (ELISpot) was performed to analyze the cellular immune responses elicited against 4 different tumor neoepitopes described for CMT64.6 (*Ndufs1*, *Itgav*, *Arghef10a.2*, and *Cep192A*), as well as against one viral protein (E1b). We detected responses against E1b and *Itgav* tumor neoepitope in VCN-01- and ICO15K-E1aPH20-treated groups (Figure S5), but responses were heterogeneous among mice, and statistical significance as a group was not achieved (Figure 3B).

No differences in immune responses were observed between VCN-01- and ICO15K-E1aPH20-treated animals; consequently, we grouped all animals for correlation analyses. There was a significant positive correlation between the number of interferon-γ (IFN-γ) spots against E1b and Itgav (Figure 3C), suggesting that anti-viral immune responses collaterally also induced anti-tumoral immune responses. Nevertheless, the immune response against *Itgav* did not correlate with tumor growth control (Figure 3D). Other tumor neoepitopes not evaluated in this study may be contributing to the antitumor response. Considering that the only difference between treatments was the hyal activity, these results are supportive of PH20 expression being responsible for the enhanced efficacy.

### Role of hyal in enhancing T cell accumulation

We aimed to analyze the relevance of hyal expression within the tumor and the immune response in a more controlled experimental system. To focus on the role of hyal in T cell infiltration separately from the role of the virus, we used a recombinant human soluble PH20 protein and an OAd capable of engaging T cells with the tumor through the secretion of a Bi-specific T cell engager. We previously published the generation of such an OAd, ICO15K-cBTE (formerly virus “ICO15K-cBiTE,” renamed for trademark issues), which expresses a T cell engager formed by a single-chain variable fragment (scFv) against the human CD3 present in T cells fused to another scFv against human EGFR (present in tumor cells), thereby recruiting T cells to EGFR-positive tumors.^25^

**Figure 4:**
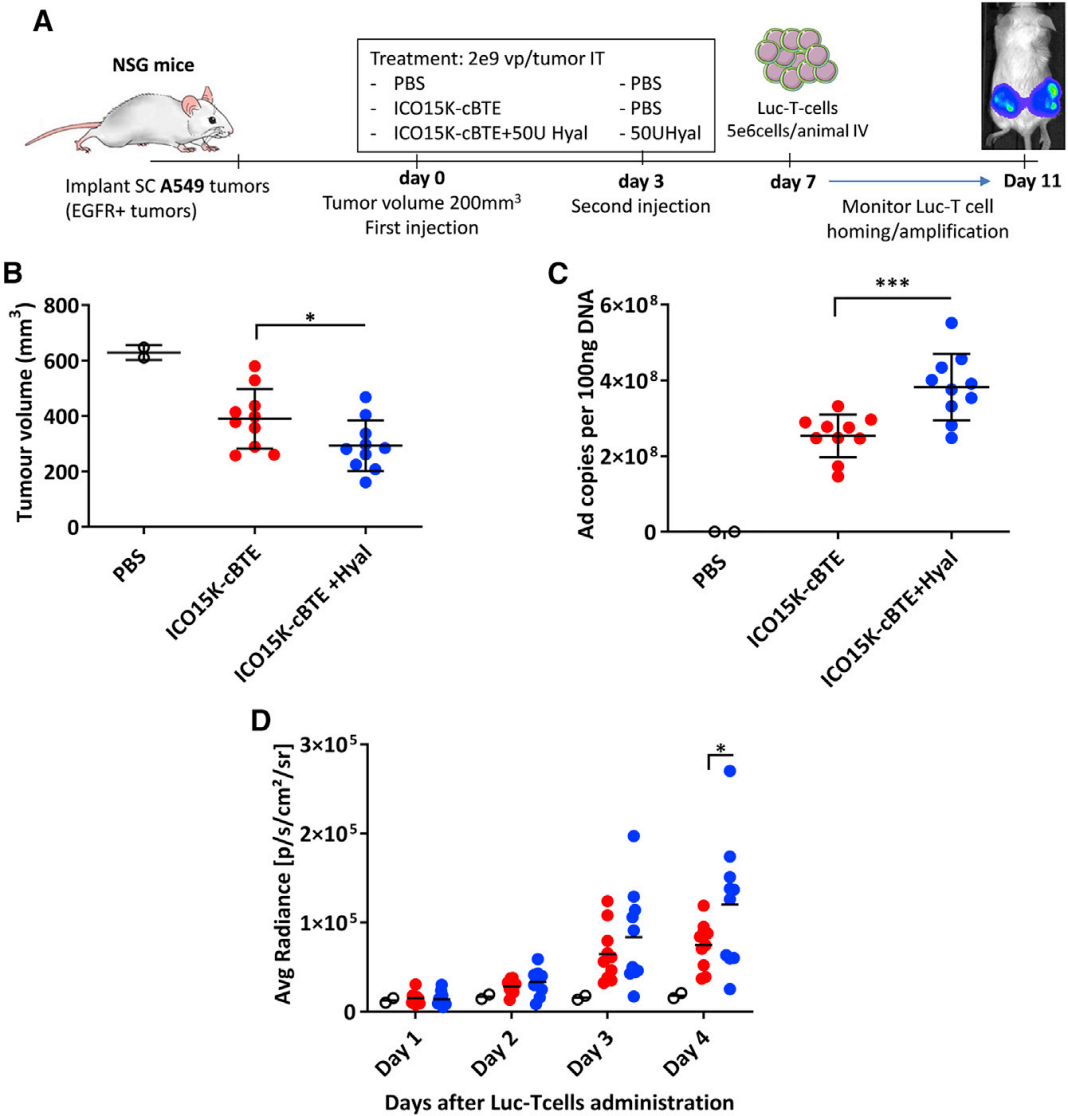
Hyal enhances T cell accumulation in tumors (A) NSG mice bearing human A549 were treated intratumorally with 2e9 vp/tumor ICO15K-cBiTE (n = 10) or ICO15K-cBiTE and 50U of human recombinant PH20 (n = 10), and then on day 3 post-treatment the hyal group was treated again with 50U hyal. (B) Tumor volume at endpoint (day 11). ∗p < 0.05 by unpaired t test. (C) The qPCR against the *E1A* gene from tumor DNA. ∗∗∗p < 0.001 by unpaired t test. (D) 4 days post final hyal injection, 5e6 luciferase-expressing T cells were injected. Bioluminescence *in vivo* was monitored for 4 days using in-vivo imaging system (IVIS). ∗p < 0.05 by RM-two-way ANOVA and Sidak’s post hoc test.

We wanted to evaluate the relevance of HA in the tumor as a barrier for T cell penetration and the role of hyal activity in allowing T cell recruitment and local amplification. Therefore, NSG mice bearing EGFR-positive (A549) subcutaneous tumors were intratumorally treated with ICO15K-cBTE as monotherapy or combined with recombinant human hyal. 3 days following treatment, tumors were injected with PBS (virus monotherapy) or a second dose of hyal (virus/hyal combination). 7 days after the first treatment, luciferase-expressing T cells were intravenously infused and monitored for 4 days by *in vivo* imaging system (IVIS, Figure 4A) to assess homing and local amplification in the tumor.

At the endpoint (day 11), the tumor volume of the virus/hyal combination group was significantly lower than that for the group treated with virus alone (Figure 4B). The number of adenovirus genomes in the tumors treated with hyal was higher than those treated only with ICO15K-cBTE (Figure 4C). The injected luciferase-expressing T cells were detected by luminescence emission in the tumors. Luminescence increased substantially in both treated groups 4 days post-T cell administration. However, tumors treated with ICO15K-cBTE combined with hyal presented higher luminescence at the endpoint than in tumors treated with virus alone. These results support the role of hyal in enhancing T cell recruitment and local amplification in established solid tumors (Figure 4D).

**Figure 5:**
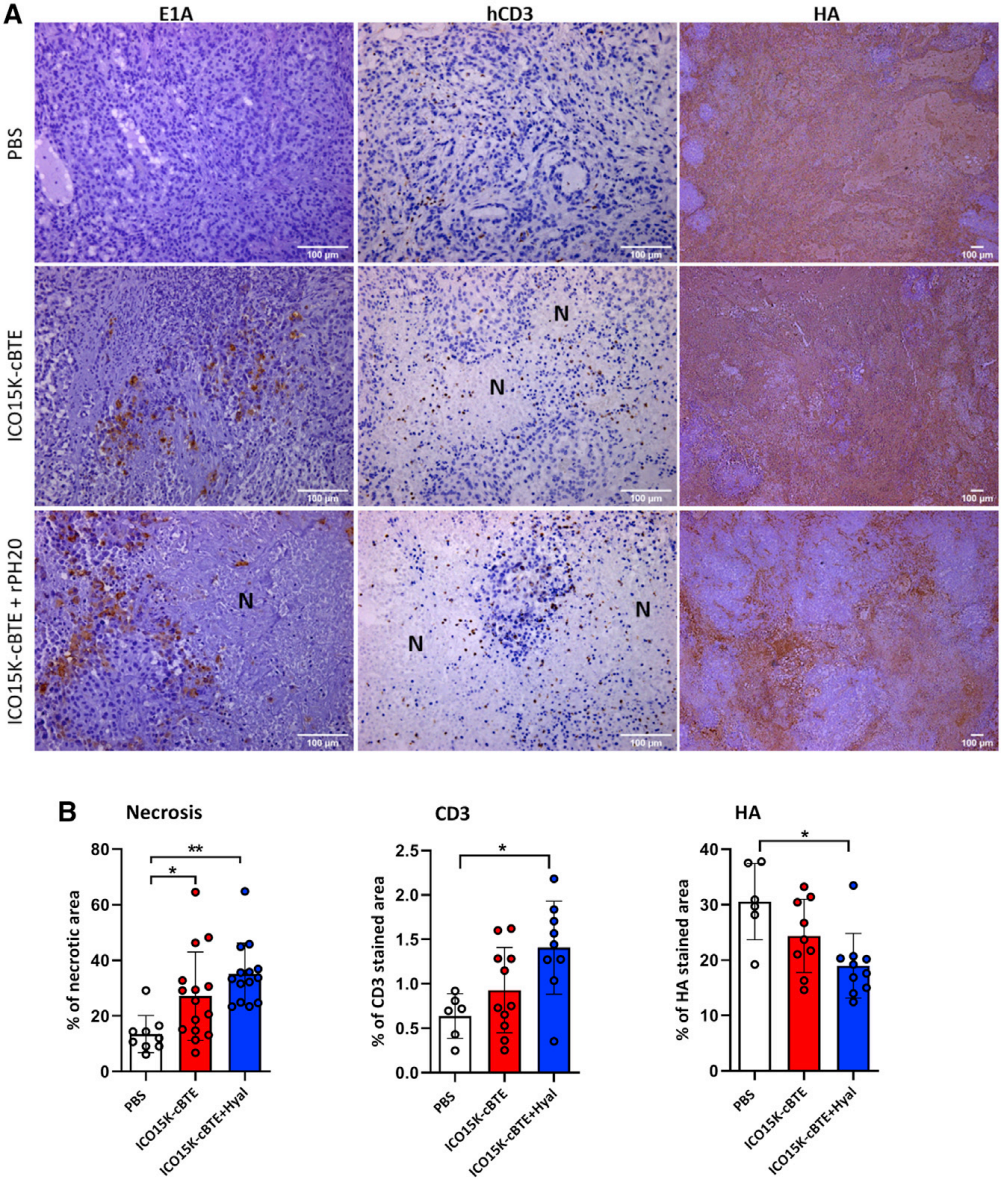
ICO15K-cBTE antitumor effect via oncolysis and T cell engagement (A) Immunohistochemistry (IHC) against viral protein (E1A, left column), infused T cells (hCD3, central column), and hyaluronic acid (HA, right column). Necrotic areas are indicated with (N). Representative images from each group are presented. (B) Random tumor areas (n > 6) were quantified for the % of necrosis (left), hCD3 staining (center), and HA (right) using FIJI software. ∗p < 0.05, ∗∗p < 0.01 by one-way ANOVA and Tukey’s post hoc test.

Tumors were also assessed by immunohistochemistry (IHC), confirming the presence of virus (E1A) in all treated groups (Figure 5A, left). Virus foci were surrounded by necrotic areas proving its oncolytic activity *in vivo*. The infused human lymphocytes were detected in each group by hCD3 staining. Tumors treated with ICO15K expressing the T cell engager showed a higher percentage of hCD3 staining and non-viable areas around T cells (Figure 5A, center), suggesting their contribution to the treatment’s antitumor effect. Hyal was not sufficiently detected (data not shown), and no significant differences were observed in the collagen fibers staining (Masson staining, Figure S6). In contrast, the combination of virus and rPH20 yielded decreased HA staining compared to other groups (Figure 5A, right, and 5B). Furthermore, more extensive necrotic areas and hCD3 staining were found in rPH20-treated tumors (Figure 5B), supporting hyal activity as an enhancer of T cell recruitment, local amplification, and intratumoral spreading of T cells and virus.

## Discussion

HA has been identified as an obstacle for cancer therapies^7^ including OVs, hampering drugs’ extravasation and intratumoral spreading.^8^ An OAd expressing hyaluronidase, VCN-01, enhanced the spread of tumor lysis in several relevant models,^15^^,^^17^ which led to the testing of VCN-01 in clinical settings.^16^ We hypothesized that a virus expressing higher amounts of hyal (PH20) might improve antitumor efficacy and the translational relevance of a hyaluronidase-expressing adenovirus.

As proof of concept, we generated a panel of viruses, which demonstrated that the insertion site downstream of *E4* offers a poor transgene expression, in line with previous results.^26^^,^^27^ In contrast, the splicing acceptor of Adenovirus 40 long fiber (40SA) produced considerably high amounts of transgenein late, thereby increasing the hyal activity of VCN-01. Furthermore, linking PH20 expression to the viral *E1A* also offered higher activity compared to VCN-01. The novel viruses ICO15K-40SAPH20 and ICO15K-E1aPH20 showed comparable cytotoxicity and production yields to VCN-01, confirming that enhanced PH20 expression did not hamper their oncolytic properties. It is worth mentioning that the P2A-virus production yields were the lowest after one replication round, though this was not significant. However, this could have an influence after several rounds of replication, in line with published P2A-armed oncolytic adenoviruses,^28^^,^^29^ at least in the A549 cell line.

We demonstrated that in a HA-sensitive model, such as Sk-mel-28,^21^ enhanced hyal expression increases antitumor activity with impressive long-term tumor growth control and significant regressions in immunodeficient mice. VCN-01 has already been proven to show an antitumor effect in less sensitive models, including even in patients. Although further studies should be performed with the novel viruses, we speculate that they would likely maintain the efficacy of VCN-01, as viral replication is not significantly affected by enhanced hyal activity.

However, with the aim of understanding the relevance of hyal activity in an immunocompetent model, we tested a high hyal-expressing virus (ICO15K-E1aPH20) and minimally hyal-expressing virus (VCN-01) in an Ad replication-restricted murine context. It is known that late genes and transgenes of human adenovirus are not translated in murine cells,^23^ as we confirmed in CMT64.6 model. The intratumoral administration of VCN-01 did not show any antitumor activity, as previously published.^30^ In contrast, ICO15K-E1aPH20 delayed tumor growth. Elevated immune responses against the virus (anti-E1B) correlated with a greater antitumor response (anti-*Itgav*), suggesting that the two immune responses are associated, as previously indicated by pre-clinical^31^^,^^32^ and clinical data.^33^ In spite of this, only the virus expressing high levels of PH20 showed efficacy, highlighting this feature as crucial for antitumor activity in this model. It should be pointed out that the immune analysis is not exhaustive, and responses against other neoepitopes might be possible. However, different neoepitope responses between viruses being the driving force of the differential activity seems unreasonable as the capsid and most of the genes are identical. Nonetheless, PH20 expression is the only variation between viruses; thus it should be contemplated that expressing a human protein within the murine tumor might induce some additional immune response. However, the virus itself generates a strong immune induction, and human PH20 has 60% homology with murine PH20. Therefore, an extra-immune response against human PH20-mediating tumor efficacy seems unlikely. Perhaps a more reasonable explanation highlights the role of PH20 activity in immune response leading to the antitumor activity. Data supporting this assumption have been published, demonstrating that pericellular HA impedes the lymphocyte-mediated cytolysis, NK recognition, and the binding of therapeutic antibodies to their targets.^34^, ^35^, ^36^

In pursuit of a more controlled system, we used a previous experimental setting published by our group.^25^ We combined an adenovirus expressing a T cell engager to promote T cell infiltration with two intratumoral PH20 injections, to degrade the HA within the tumor and evaluate whether HA is a barrier for T cell infiltration. As expected, we observed an enhanced antitumor efficacy in combination with hyal;^37^ more viral genomes were detected in tumors treated with hyal in addition to increased T cell accumulation, proposing for the first time that hyal activity increases T cell infiltration and local spreading. Virus and T cells were localized surrounding necrotic areas, proving their involvement in the antitumor effect. Despite the lack of hyal detection, likely due to its short half-life,^38^ its effect is evidenced by the lack of HA, extended necrotic areas, and lower tumor volumes with the combined treatment.

Several promising immunotherapies such as CAR T cells, adoptive cell transfer, and immune checkpoint inhibitors have shown limited efficacy in “cold” solid tumors. Given that oncolytic adenoviruses are capable of inducing anti-virus and antitumor immune responses and that hyal activity increases both the oncolytic antitumor effect and T cell accumulation, we propose that a virus with a high-hyal activity could be a suitable agent for combinations with other immunotherapies, especially CAR T cell therapy.

## Materials and methods

### Cell lines

The cancer cell lines A549, MDA-MB-231, HT1080, MIA PaCa-2, and FADU were obtained from the American Type Culture Collection (ATCC, Manassas, VA, USA). The CMT.64 cell line was provided by Dr. Stephan Kubicka (Hannover Medical School, Hannover, Germany), and the most permissive clone to adenovirus infection was previously isolated and expanded by our group, generating the CMT64.6.^24^ All cell lines were maintained with DMEM supplemented with 10% FBS (GIBCO) and maintained at 37°C, 5% CO_2_.

### Recombinant adenoviruses

ICO15K^18^ and VCN-01^19^ were previously described. The new recombinant adenoviruses were generated by homologous recombination in bacteria using PCR fragments, as described.^39^^,^^40^ Briefly, the PCR fragment of the desired modification (primers available by request) was introduced in electrocompetent bacteria containing a bacterial artificial chromosome, which encodes for the ICO15K backbone with a selection gene in the desired location. Once recombined, the new plasmid was transfected into HEK293 cells, and the resulting virus was amplified in successive rounds in A549 cells and purified on a CsCl gradient according to standard protocols.

### Virus cytotoxic assays

Virus cytotoxicity assays were performed as previously described.^18^ Briefly, a serial dilution of viral TU was used to infect the desired cell line in triplicate. The initial multiplicity of infection (MOI; TU/cell) and the number of cells should be carefully adjusted depending on the cell line. After 4 days of infection, the cell viability was assessed by bicinchoninic acid assay (BCA, Pierce Biotechnology). Absorbance was quantified, and the number of TU per cell required for 50% inhibition (IC_50_) was estimated from a dose-response non-linear regression with a variable slope, calculated with GraphPad Prism v6.02 (GraphPad Software).

### Production assay

A549 (10^5^ cells) or CMT64.6 (3 × 10^5^ cells) were seeded in 24-well-plates and incubated overnight. Cells were infected with the parental virus and tested viruses at MOI of 20 (A549) or 400 (CMT64.6), in 500 μL of DMEM 5% FBS for 4 h. Then cells were washed once with PBS and fed with fresh media. CEs and SNs were collected over desired time points and were prepared by 3× freeze/thaw cycles. The functional titer was determined with the anti-hexon staining method.^41^ Results are expressed as the number of TU produced by a single cell, considering the functional titer and the number of cells on the day of the infection.

### Turbidimetric assay

The turbidimetric assay protocol was previously described.^19^ SNs of infected cells were harvested and concentrated 20-fold with Amicon Ultra-15 filter units with a molecular weight cutoff of 30 kDa (Merck Millipore) or not, as indicated. SNs were mixed with HA (Sigma) at 0.03% w/v solution in phosphate buffer (300 mN sodium phosphate monobasic, pH = 5.35 at 37°C) and incubated overnight (12–18 h) at 37°C. The next day, the reaction was stopped by adding 5 volumes of acid albumin solution (24 mM sodium acetate, 79 mM acetic acid, and 0.1% of bovine albumin [pH = 3.75]), and the absorbance at 600 nm was measured. Low absorbances indicate low quantity of HA.

### *In vivo* antitumor efficacy

The *in vivo* studies were performed at Biomedical Research Institute of Bellvitge (IDIBELL) facility (AAALAC unit 1155) and approved by the Ethics Committee for Animal Experimentation off IDIBELL. 6- to 8-week-old NOD/*scid*/*IL2rg*^*–/–*^ (NSG) female mice (bred in house) were implanted with Sk-mel-28 subcutaneous tumors. Animals were randomized into treatment groups (n ≥ 7 tumors per group) when tumors reached a mean of 180 mm^3^. Mice were treated with an intravenous injection of 2 × 10^10^ vp/animal in 200 μL of PBS, and tumor volume was monitored for 81 days. At the endpoint, tumors were collected for IHC analyses. 6-week-old female C57BL/6J mice (Charles River) were implanted with CMT64.6 tumors (subcutaneous) in both flanks. Mice were randomly allocated to groups (n = 10 tumors per group) when tumor volume average reached 80 mm^3^. Then tumors were injected at 1 × 10^9^ TUs in 30 μL of PBS. Tumor volume was monitored for 18 days and mice were sacrificed to isolate splenocytes for ELISpot.

### ELISpot

Lymphocyte-specific responses were evaluated by anti-IFN-γ ELISpot according to standard protocols, as published by our group.^42^ For CMT64.6 neoepitopes the peptides of *Nduf1s* (AAVSNMVQKI), *Arghef10a.2*, (AAVKRGRSFI), and *Cep192A* (QIINNSVTL) were used based on previous publications.^30^^,^^43^ The *Itgav* (SSILYVKSL) was predicted *in silico* by NetMHCcons v1.0. For antiviral response, E1b_192_ (VNIRNCCYI) was used. All peptides were produced by JPT Innovative Peptide Solutions (Germany).

### IHC

Paraffin-embedded blocks were cut into 4-μm thick sections. E1A and HA staining were performed as previously published,^14^^,^^21^ using the anti-Ad2/5 E1A antibody (1/200; Santa Cruz Biotechnology, SC-25) and B-HABP (5 μg/mL; Amsbio, AMS.HKD-BC41). For human CD3 detection, FLEX Polyclonal Rabbit Anti-Human CD3 (IR503, Agilent DAKO) was diluted 1:10 with DAKO antibody diluent (Dako – Agilent, S0809) for 120 min at room temperature. The secondary antibody used was a BrightVision poly-horseradish peroxidase (HRP)-anti-rabbit immunoglobulin G (IgG) that was biotin-free, ready to use (Immunologic, DPVR-110HRP) incubated 45 min. Antigen-antibody complexes were reveled with 3-3′-diaminobenzidine (K3468, Dako). H&E staining was performed according to standard procedures. Masson trichromic staining was performed using the Accustain Trichrome Stain Kit (Sigma Aldrich) according to the manufacturer’s indications. The percentage of stained areas in IHC images were quantified after color deconvolution by ImageJ FIJI^44^ software.

### T cell accumulation *in vivo*

6- to 8-week-old NOD/*scid*/*IL2rg*^*–/–*^ (NSG) female mice (bred in house) were implanted with A549 tumors subcutaneously (2 tumors per animal), and then animals were randomized into treatment groups (n = 10 tumors per group) with a mean tumor volume of 210 mm^3^. Mice were treated with an intratumoral injection of 2 × 10^9^ vp/tumor w/o 50U of rPH20 (Acro Biosystems, PH0-H5225) in 30 μL of PBS. 3 days post-treatment, 50U of rPH20 or PBS (30 μL) was injected into each tumor according to treatment group. 4 days later (7 days from first treatment), pre-activated GFP- and CBG-luciferase-expressing T cells (Luc T cells) were intravenously administered (5 × 10^6^ cells/animal in 200 μL) with an intraperitoneal injection of hIL-2 (1,500 U/animal, Proleukin). Mice were given an intraperitoneal injection of 15 mg/mL D-luciferin potassium salt solution (Byosinth AG) and imaged daily for 4 days using Lumina XRMS Imaging System (IVIS, PerkinElmer). Tumor luminescence was measured by selecting the tumor contour and subtracting the background.

### Virus detection in tumors

Tumors were homogenized with UPHO homogenizer (Geneye, 77.GY-U001). Total DNA was extracted from 15 μL of homogenized tissues using QIAamp DNA Mini kit (QIAGEN, 51306). VCN-11 viral genomes in total DNA were quantified by qPCR using the Light Cycler 480 II system (Roche), and Ad18852F (5′-CTTCGATGATGCCGCAGTG-3′) and Ad19074R (5′-ATGAACCGCAGCGTCAAACG-3′) primers.

### Statistical analysis

Statistical analyses were performed using Graphpad Prism Software v8.0. All results are expressed as means ± SD or SEM, as indicated. p value < 0.05 was used as the threshold for significance. First, data were assessed for normality by Shapiro-Wilk test and depending on the result parametric or non-parametric tests were performed, as indicated in each figure. For *in vivo* studies, a mixed two-way ANOVA of repeated measures (Graphpad Prism) was used to compare the means between all groups for each measurement. Correlations of parametric data were assessed using Pearson correlations, whereas Spearman correlations were used for non-parametric data.

## Supplemental information

Document S1. Table S1 and Figures S1–S6

Document S2. Article plus supplemental information
