# Supplementary material for: Hyaluronidase expression within tumors increases virotherapy efficacy and T cell accumulation
Source: Mol Ther Oncolytics. 2021 May 29;22:27–35. doi: 10.1016/j.omto.2021.05.009 (PMC8321894; doi:10.1016/j.omto.2021.05.009)
Supplement: Document S1. Table S1 and Figures S1–S6 [file mmc1.pdf]

OMTO, Volume *22*

Supplemental information

Hyaluronidase expression within tumors increases virotherapy efficacy and T cell accumulation

Martí Farrera-Sal, Rafael Moreno, Ana Mato-Berciano, María Victoria Maliandi, Miriam Bazan-Peregrino, and Ramon Alemany

SUPPLEMENTARY MATERIAL

Supplementary Table 1. Titers of purified viruses. Vp: viral particles, TU: transfecting units.

Purified viruses	Physical Titer (vp/mL)	Function Titer (TU/mL)	Vp/TU Ratio
ICO15K	3,93E+12	4,92E+11	8,0
VCN-01	1,18067E+12	7,83E+10	15,1
ICO15K-40SAPH20	4,150E+12	3,75E+11	11,07
ICO15K-E1aP2APH20	4,600E+12	4,18E+11	11

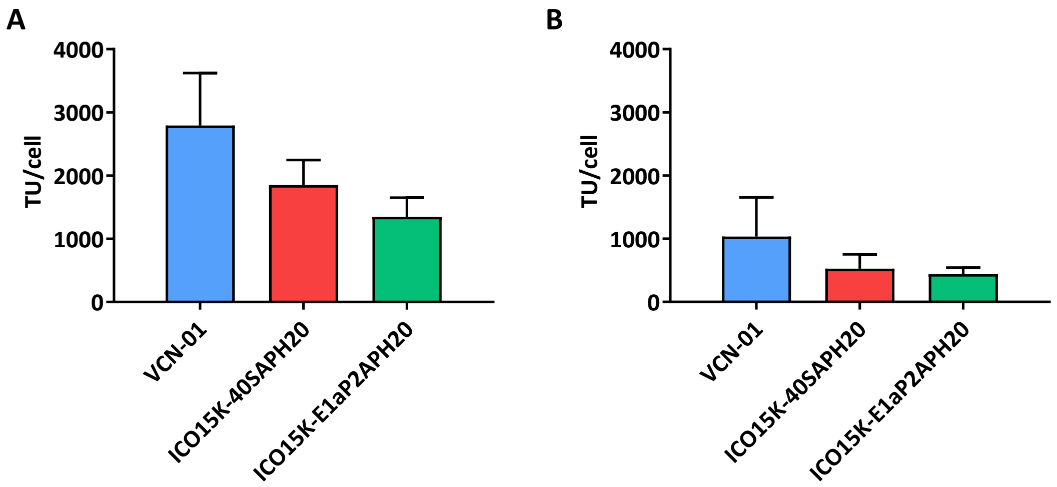

Supplementary Figure 1. Virus production yields. A549 cell line was infected at MOI 20 for 4 hours. Then, excess of viruses was washed and cells were incubated for 24, 48 or 72 hours. Total virus production at 72hours is detailed in **(A)** culture cell extracts (CE) or in **(B)** supernatants (SN), which was determined by anti-hexon staining method. Results are expressed as transducing units (TU) produced per cell. Mean±SD of triplicates is shown. *$p<0.05$ by Kruskal-Wallis with Dunn's post-hoc test.

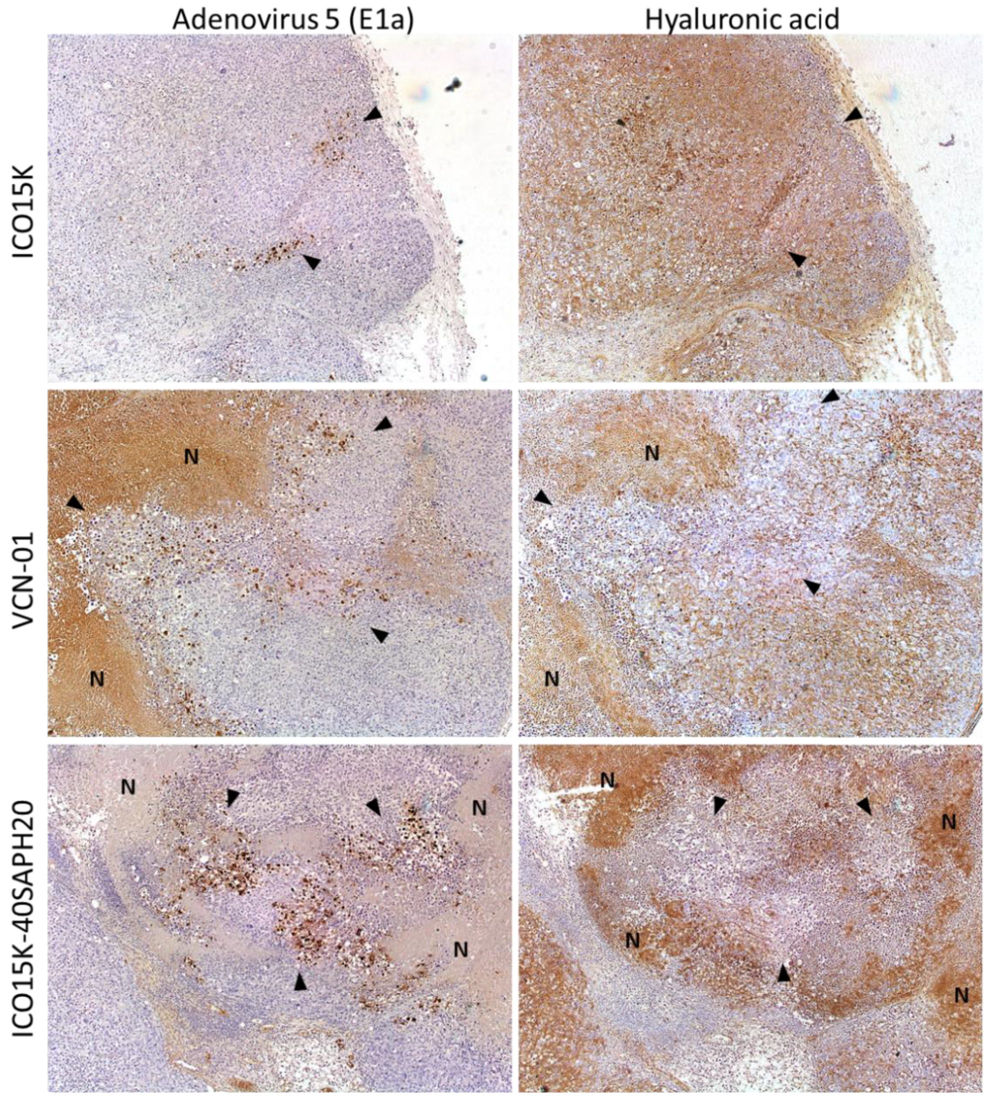

Supplementary Figure 2. Hyaluronidase activity *in vivo*. Immunohistochemical staining of E1A viral protein (left column) and hyaluronic acid (right column) were performed in Sk-mel-28 tumors after 40 days post-treatment with $1x10^8$ TUs of ICO15K (first row), VCN-01 (second row), and ICO15K-40SAPH20 (third row). Representative images at 40x were presented with black arrows indicating viral replication focus. Necrotic zones were unspecifically stained, so should not be considerate positive in the IHC evaluation (marked with an N in the images).

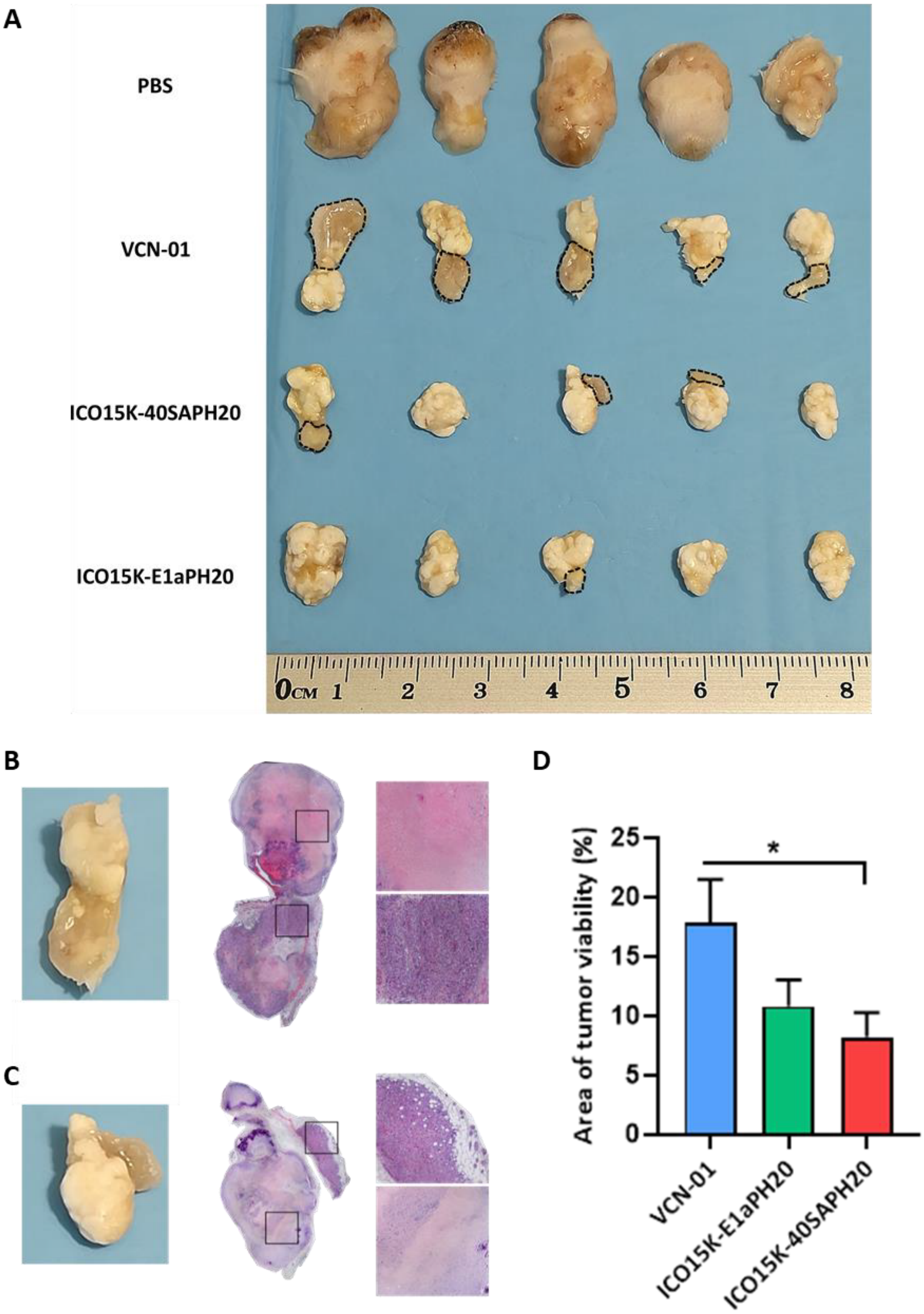

Supplementary Figure 3. Macroscopic images of Sk-mel-28 tumors treated with Hyal-OAds. NSG mice bearing Sk-mel-28 tumors were treated intravenously with different viruses. Control group (PBS) was sacrificed according to ethical criteria on day 44 after treatment. The endpoint for the VCN-01, ICO15K-40SAPH20, and ICO15K-E1aPH20 was on day 81 post-treatment. **(A)** Viable growing nodules were detected macroscopically and highlighted in the image with dotted black line. Most of tumors ICO15K-40SAPH20 and ICO15K-E1aPH20 were highly necrotic. Tumors treated with **(B)** VCN-01 or **(C)** ICO15K-40SAPH20 were analyzed macroscopically (left), microscopically by IHC of hematoxilyn and Eosin (center), and non-cellular zones (red staining) and tumor cells (blue) were identified (right). **(D)** The percentage of viable tumor (nucleus integrity, blue staining) was quantified by FIJI/ImageJ Software. *$p<0.05$ by Kruskal-Wallis and Dunn's *post hoc* test (n=5).

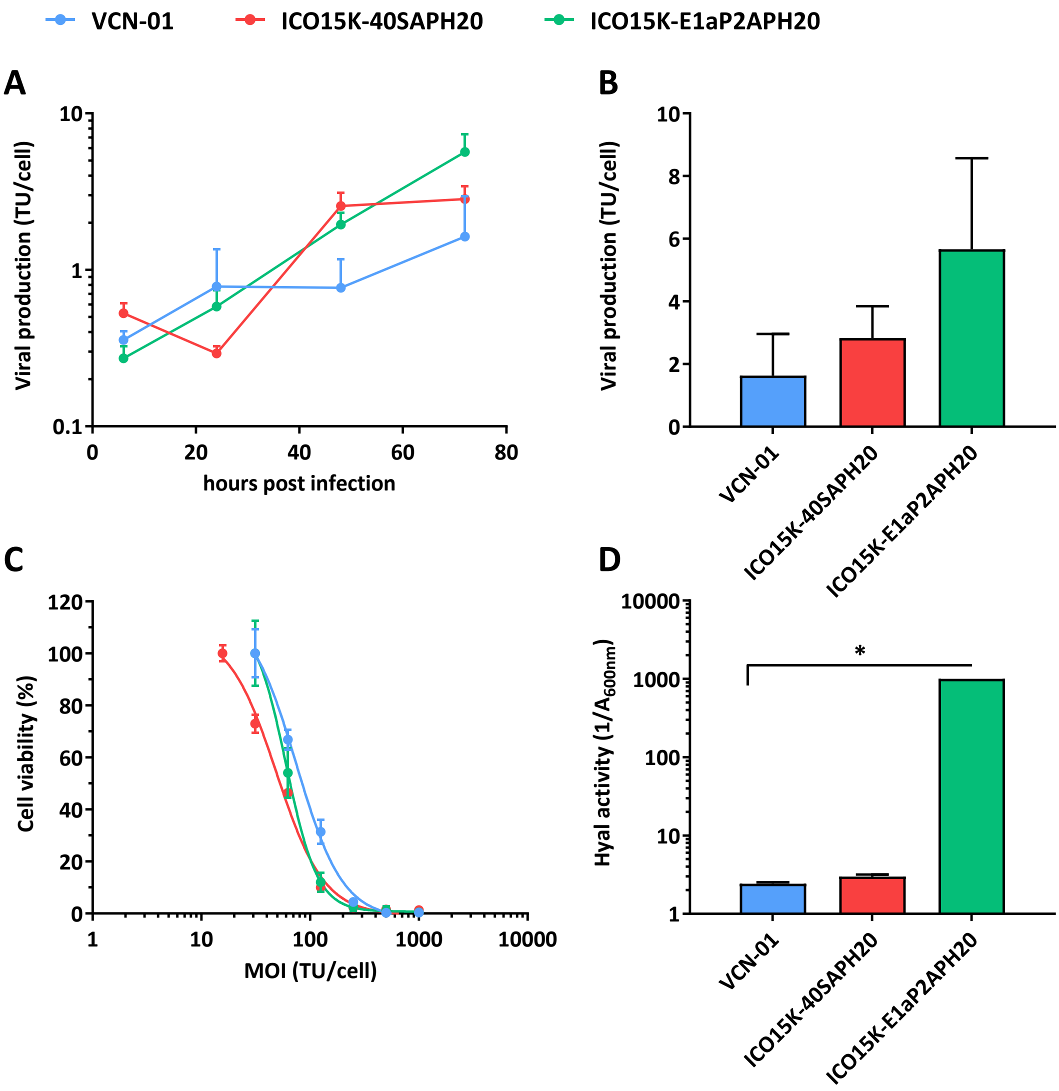

Supplementary Figure 4. *In vitro* characterization of Hyal-OAds in CMT64.6 murine model. (A) CMT64.6 cell line was infected at MOI 400 for 4 hours. Then, excess of viruses was washed, and transfecting units production was assessed at 24h, 48h and 72h. **(B)** The last timepoint is detailed. Total virus production in culture cell extracts was determined by anti-hexon staining method. Results are expressed as transducing units (TU) produced per cell. Mean ± SD of triplicates is shown. No significant differences were observed by Kruskal-Wallis with Dunn's post hoc test. **(C)** CMT64.6 cells were infected, ranging from 1000 TU/cell to 0 TU/cell and 5 days post-infection cell viability was determined by BCA staining. The IC50 for each virus was calculated. **(D)** A turbidimetric assay was performed with supernatants harvested at 72hours post-infection of CMT64.6 cells at MOI 400. The hyaluronidase activity is represented as the inverse of the absorbance at 600nm (1/A600nm). Mean±SD of triplicates is plotted. *$p<0.05$ by Kruskal-Wallis with Dunn's post hoc test.

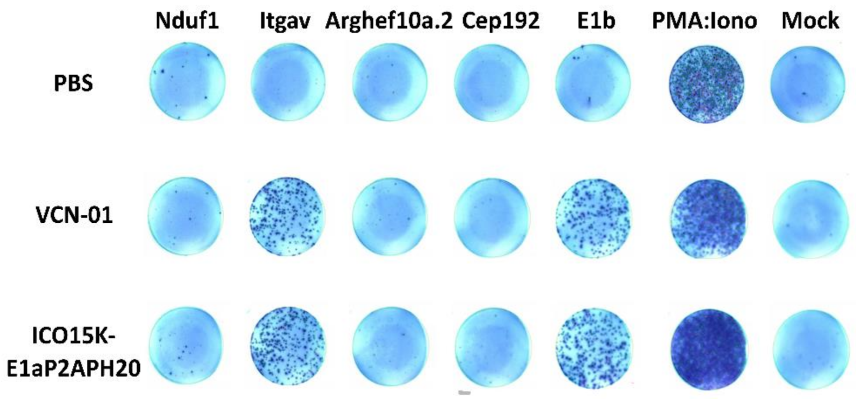

Supplementary Figure 5. Immune response in CMT64.6 treated tumors. Representative images ELISpot wells for every group. PMA:Iono was used as a positive control of stimulation, and Mock as a negative control.

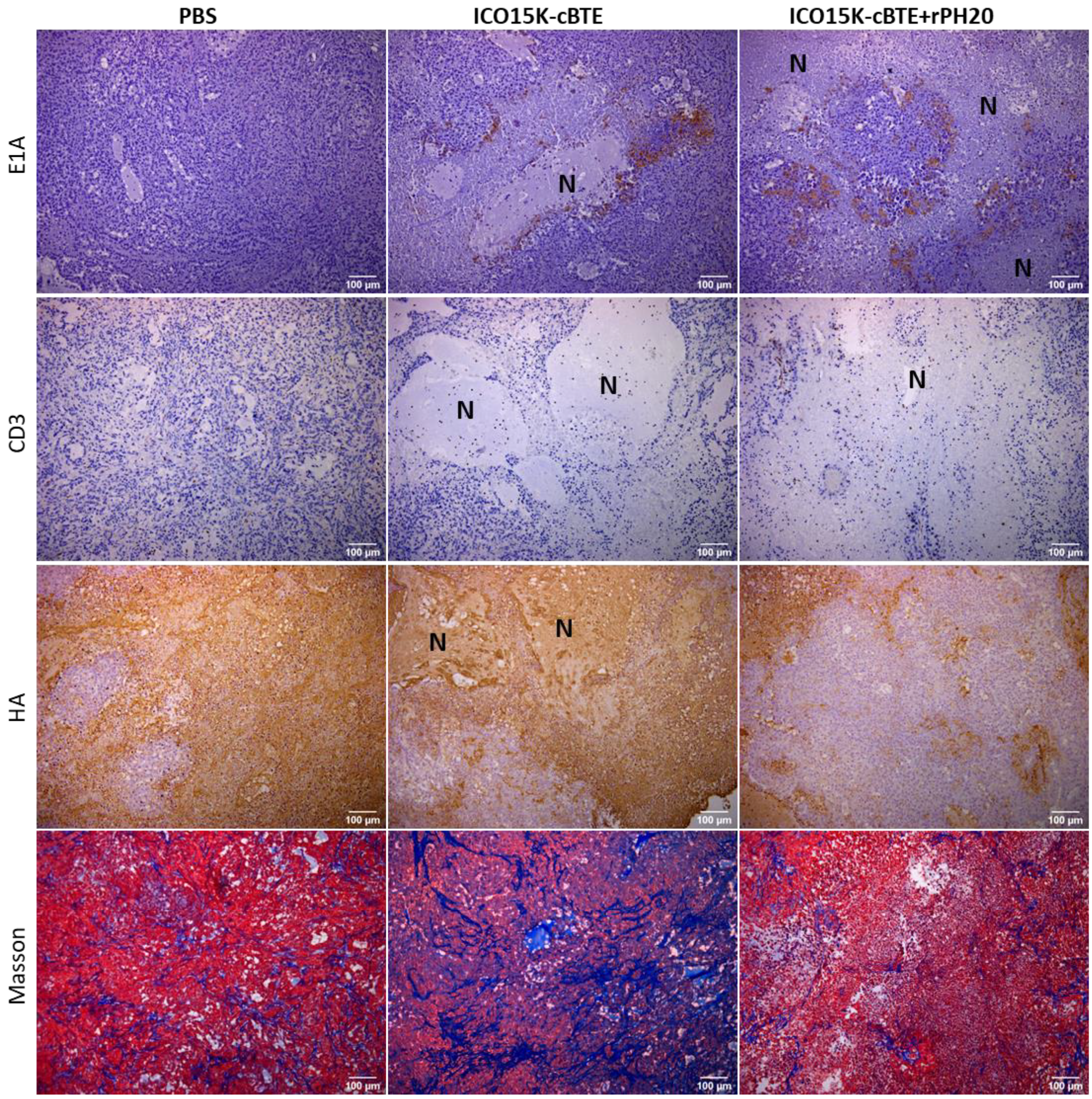

Supplementary Figure 6. Effect of ICO15K-cBTE and rPH20 treatment. Immunohistochemistry against viral protein (E1A, first row), lymphocytes (hCD3, second row), hyaluronic acid (HA, third row), and collagen fiber (Masson staining, fourth row). Necrotic areas are indicated by an N. Representative images of each group are presented.
